# Secretoneurin levels are higher in dilated cardiomyopathy than in ischaemic cardiomyopathy: preliminary results

**DOI:** 10.3389/fcvm.2023.1297900

**Published:** 2024-01-08

**Authors:** Jiří Plášek, Jozef Dodulík, Marie Lazárová, David Stejskal, Zdeněk Švagera, Nela Chobolová, Patrik Šulc, Lukáš Evin, Dana Purová, Jan Václavík

**Affiliations:** ^1^ Department of Internal Medicine and Cardiology, University Hospital Ostrava, Ostrava, Czechia; ^2^ Research Center for Internal and Cardiovascular Diseases Faculty of Medicine, University of Ostrava, Ostrava, Czechia; ^3^ Institute of Laboratory Medicine, University Hospital Ostrava, Ostrava, Czechia; ^4^ Institute of Laboratory Medicine, University of Ostrava, Ostrava, Czechia; ^5^ Social Health Institute, Palacky University Olomouc, Olomouc, Czechia

**Keywords:** secretoneurin, heart failure, CaMKII, dilated cardiomyopathy, ischaemic cardiomyopathy

## Abstract

**Background:**

Secretoneurin (SN) is a neuropeptide with potential utility as a biomarker of cardiovascular episodes. The main effect of SN is mediated through its inhibition of calmodulin-dependent kinase II (CaMKII), which influences calcium handling. We aimed to associate the levels of SN in plasma with different causes of heart failure.

**Methods:**

We prospectively enrolled consecutive patients with ischaemic (ICM) and dilated (DCM) cardiomyopathy from the outpatient heart failure clinic and healthy individuals. SN was analysed from venous blood by use of the ELISA method. SN plasma levels were compared in DCM, ICM and healthy individuals with non-parametric tests.

**Results:**

A total of 53 patients (81.1% male, 18.9% female; mean age 67.9 ± 12.6 years) and 34 healthy individuals (38% male, 62% female) were included in the analysis. Plasma SN levels were significantly higher in the dilated cardiomyopathy (38.8 ± 27 pmol/L) as compared with the ischaemic cardiomyopathy (19.7 ± 22.6 pmol/L) group (*P* = 0.006). There was no significant difference between females vs. males (27.1 ± 23 vs. 25.5 ± 26.2 pmol/L, *P* = NS). Plasma SN levels allowed DCM and ICM to be differentiated with 88% sensitivity and 61% specificity (*P* = 0.007), the cut of value is 13.3 pmol/L. Plasma SN levels differed significantly between healthy volunteers and both ICM (*P* < 0.0001) and DCM (*P* = 0.049). Plasma SN levels did not differ according to age and were not associated with comorbidities, left ventricular ejection fraction, heart failure medication, troponin, creatinine, or natriuretic peptide plasma levels.

**Conclusion:**

Plasma secretoneurin levels differed significantly in DCM vs. ICM, being higher in the former. Based on plasma SN levels, discrimination between DCM and ICM might be possible. Healthy individuals produce higher SN plasma levels than stable HFrEF patients.

## Graphical Abstract

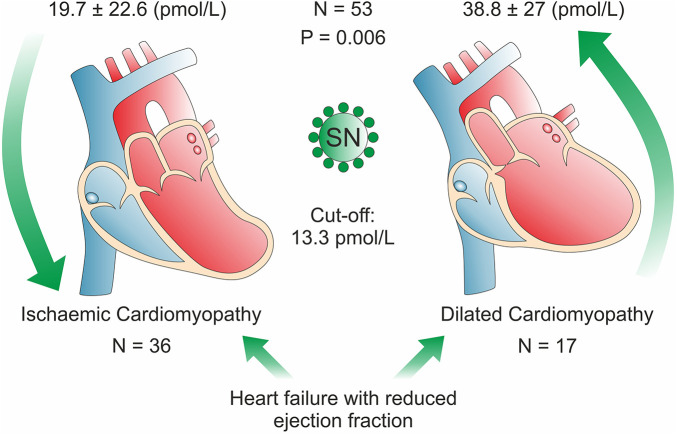

## Introduction

Secretoneurin (SN) is a 33–amino acid neuropeptide from the chromogranin peptide family. SN may be a novel biomarker with potential use in cardiovascular medicine (1). Its pathway differs from those of the most often measured natriuretic peptides and troponin. SN's main effects are most likely transmitted by calmodulin-dependent kinase II (CaMKII). However, other cellular pathways may play a role (2). Since CaMKII is one of the regulators of calcium handling in the cell, it may enhance protective effects of SN in the diseased myocardium (3). CaMKII inhibition in the myocardium improves contraction and suppresses proneness to arrhythmia by diminishing calcium leakage from the sarcoplasmic reticulum (3). Calcium is crucial to myocardial excitation-contraction coupling and intracellular signalling. Since in heart failure patients, the calcium handling is known to be disrupted, the SN plasma levels may entail prognostic information (4).

Of note, in the recent sub-analysis of the GISSI-HF trial in patients with chronic heart failure, SN concentrations were associated with mortality, even after adjusting for multiple factors (5). SN levels were also weakly associated with admission to the hospital for cardiovascular reasons (5).

We already know that SN levels may contain prognostic information in heart failure (HF) patients. Therefore, we aim to address possible differences in the SN levels associated mainly with the HF aetiology.

## Materials and methods

### Patients

For this study, we prospectively enrolled 53 consecutive patients from the heart failure outpatient clinic of University Hospital Ostrava from August 2022 to January 2023 and 34 heathy volunteers in October 2023. The study sample comprised only patients with a reduced ejection fraction of two causes (ischaemic cardiomyopathy, dilated cardiomyopathy). All patients were in stabilized condition without recent heart failure decompensation. All patients with dilated cardiomyopathy have no coronary artery disease (no coronary artery stenosis ≥ 70% or 50% of the common trunk of the left coronary artery). Acute myocardial infarction and/or coronary artery by-pass grafting surgery within three months before enrolment were exclusion criteria.

The healthy volunteers were without any treated disease. Most importantly, no cardiac disease was present, defined by the absence of clinical symptoms, normal echocardiography, and normal plasma levels of markers of cardiac injury at the time of blood sampling. This study was approved by the Institutional Review Board of University Hospital Ostrava (Nr. 526/2022) and conducted in accordance with the Helsinki Declaration. All patients have signed informed consent.

### Secretoneurin analysis

Blood sampling was performed in patients with chronic stable heart failure during outpatient visits. SN was analysed from venous blood by use of the ELISA method (CardiNor AS, Oslo, Norway) (6). The blood was drawn into the lithium-heparin tubes, plasma was separated and frozen to −70°C, only one defrosting cycle was allowed. The intra-assay coefficients of variation for SN were lower than 5% and inter-assay coefficient lower than 10%. The level of determination (LoD) for the CardiNor SN is 5,1 pmol/L, The level of quantification (LoQ) is 7,6 pmol/L. Analytical range is 11,8–299,2 pmol/L.

### Statistics

Continuous variables are expressed as mean ± standard deviation or as median and interquartile range when indicated and compared by the Mann–Whitney *U* test since the data were non-normally distributed according to both the Kolmogorov-Smirnov and Levene's tests. Categorical variables are expressed as percentages and compared by the chi-square test, Fisher's exact test, or logistic regression, as appropriate. Correlations between SN levels and other biomarkers were examined by Spearman correlation. The difference between plasma SN levels in ICM vs. DCM or healthy individuals was examined by Kruskal-Wallis or Mann–Whitney *U* tests. Receiver operating characteristics analysis was performed for SN discriminative abilities related to the cause of heart failure. A two-tailed *α* < 0.05 was considered statistically significant. All analyses were performed using IBM SPSS for Mac version 23 (IBM, Armonk, USA).

## Results

A total of 53 HFrEF patients (81.1% male, 18.9% female) aged 67.9 ± 12.6 with a BMI of 27.7 ± 4.4 and 34 healthy individuals (38% male, 62% female) were included in the analysis. Mean SN values (pmol/L) according to the cause of heart failure were 19.7 ± 22.6 for ischaemic cardiomyopathy (*N* = 36), 38.8 ± 27 for dilated cardiomyopathy (*N* = 17) (7); the median and IQR were as follows ICM: 13.3(29.6), DCM: 34.4 (27.2) pmol/L. Mean SN values for health individuals were 50.7 ± 15.3 pmol/L. The mean left ventricular ejection fraction (LV EF) in HFrEF patients was 29.7 ± 6.6. Comorbidities, anthropometric factors, medication, mean levels of biomarkers and plasma electrolytes are depicted in Table 1.

**Table 1:** Baseline characteristics of the study population, ischemic (ICM) vs. Dilated cardiomyopathy (DCM).

	Total population	ICM	DCM	*P* value
	*N* = 53	*N* = 37	*N* = 16	
Age (years)	67.9 ± 12.6	69.9 ± 11.6	65.7 ± 14.6	0.574
Males (%)	81.1	83.7	70.6	0.260
Secretoneurin	25.8 ± 25.4 22.5 (45.3)	19.7 ± 22.6 13.3 (38.5)	38.8 ± 27 34.4 (27.2)	0.006
Body weight (kg)	77.1 ± 27.3	73 ± 31	85.6 ± 15.4	0.194
Body height (cm)	172.8 ± 10.8	173.3 ± 10.6	171.8 ± 11.4	0.760
Body mass index (kg/m^2^)	27.7 ± 4.5	27 ± 3.9	29.4 ± 5.5	0.084
LV ejection fraction (%)	29.7 ± 6.6	30.8 ± 6.5	27.2 ± 6.3	0.066
Hypertension (%)	86.2	54	29.4	0.052
AF (%)	8.5	8.1	5.9	1.0
Dyslipidaemia (%)	79.3	54	17.6	0.003
Diabetes mellitus (%)	34.6	32.4	35.3	1.0
Previous stroke/TIA (%)	9.6	16.2	5.8	0.193
NYHA class II (%)	57.7	59.4	47	0.111
NYHA class III (%)	34.6	32.4	35.3	0.128
ACEi (%)	34.8	29.7	29.4	1.0
sGLT2i (%)	56.5	51.3	41.2	0.527
Betablockers (%)	87	70.3	82.3	0.647
MRCA (%)	77.8	62.2	70.6	1.0
Amiodaron (%)	37.8	26.7	35.3	0.497
ARNI (%)	51.1	40.5	50	1.0
NT-pro-BNP	2876.2 ± 4709 1,376 (2885)	3346.9 ± 5539.4 1402.7 (3927)	1990.2 ± 2407 1,000 (2504.6)	0.681
Creatinine	118.3 ± 66.8	103.1 ± 33.1	101.6	0.037
Potassium	3.78 ± 1.4	3.5 ± 1.7	4.3 ± 0.43	0.115
Total calcium	1,59 ± 1.26	1.66 ± 1.26	1.48 ± 1.3	0.617
hs-TnI	526.78 ± 2112	761.7 ± 2597.9	86 ± 136.4	0.474
CRP	8.3 ± 13.8	9.7 ± 15.8	5.4 ± 7.7	0.955

Indices are shown as mean ± standard deviation or percentages for categorical variables and compared for ICM and DCM. As an alternative median and interquartile range is shown when indicated—SN, NT-pro-BNP. ACEi, angiotensin-converting enzyme inhibitor; AF, atrial fibrillation; ARNI, angiotensin receptor/neprilysin inhibitor; CRP, C- reactive protein; hs-TnI, high sensitivity Troponine I; LV, left ventricle; MRCA, mineralocorticoid receptor antagonist; NT-pro-BNP, N-terminal pro brain natriuretic peptide; NYHA, New York heart association; sGLT2i, sodium-glucose cotransporter 2 inhibitors; TIA, transient ischemic attack.

**Figure 1:**
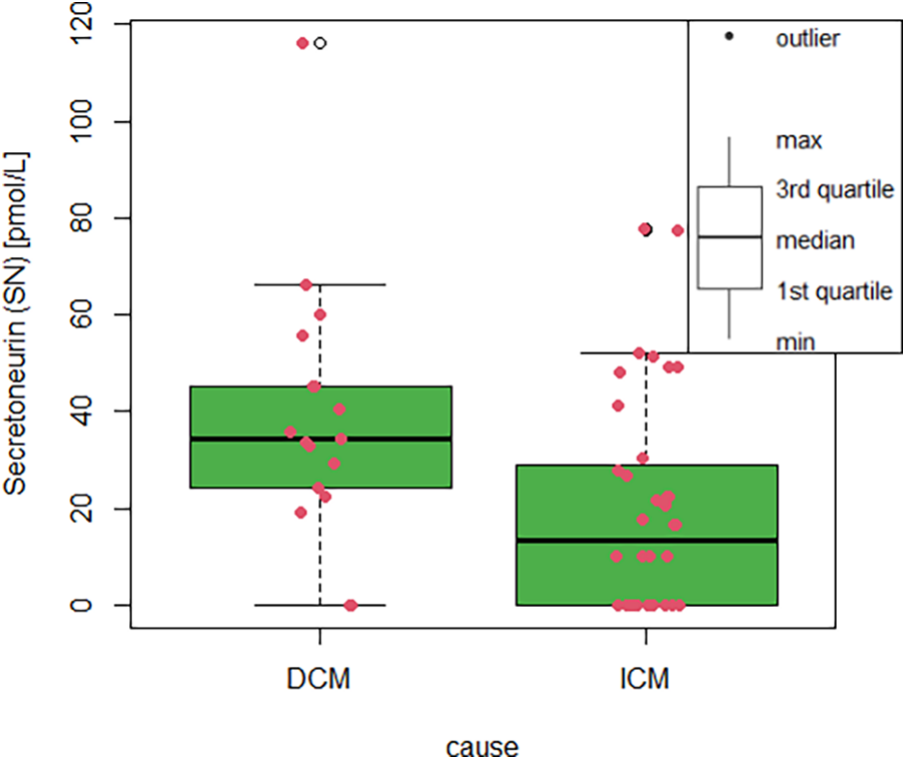
Box-plot with whiskers displaying differences in SN plasma level between ICM and DCM. The red dots stand for individual measurement value. Secretoneurin (SN) plasma levels in pmol/L according to the cause of heart failure; ICM, ischaemic cardiomyopathy; DCM, dilated cardiomyopathy.

**Figure 2:**
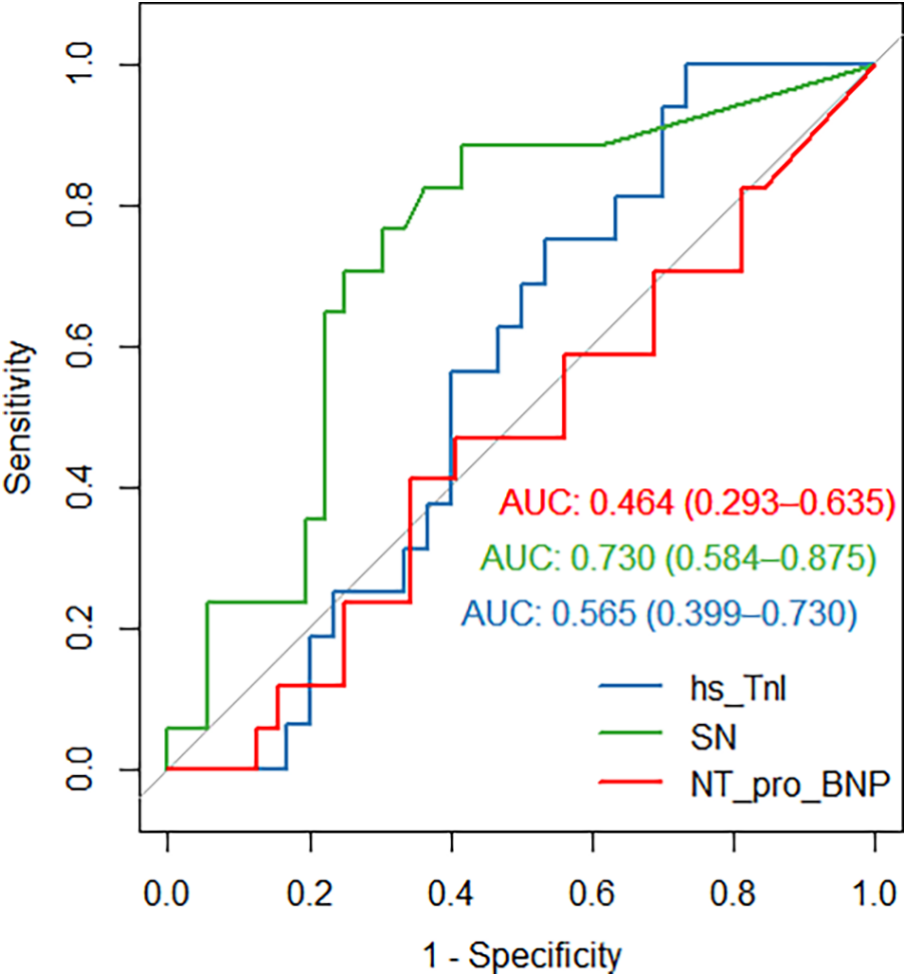
Receiver operating curve (ROC) for secretoneurin (SN, green curve), high-sensitivity troponin I (hs-TnI, blue curve) and N-terminal-pro natriuretic peptide (NT-pro-BNP, red curve) differentiating dilated cardiomyopathy from ischaemic cardiomyopathy (SN, AUC 0.73, 95% CI 0.58–0.87, *P* = 0.007; TnI, AUC 0.56, 95% CI 0.4–0.73, *P* = NS;NT-pro-BNP, AUC 0.44, 95% CI 0.27–0.62, *P* = NS).

SN plasma levels differed significantly in the dilated cardiomyopathy (DCM) as compared with the ischemic cardiomyopathy (ICM) group (*P* = 0.006, Figure 1), irrespective of NYHA class, heart failure pharmacotherapy and LV EF. In the multivariable analysis, SN still differed significantly between ICM and DCM, even after entering LV EF, age, heart failure treatment and hypertension/gender as covariates in the regression model. On the contrary, LV EF did not differ significantly between the DCM and the ICM group (27.2 ± 6.3 vs. 30.8 ± 6.5, *P* = NS). The presence/absence of outliers in both groups did not change the test result, what means that the significance or non-significance of the test was not different; the outliers stayed in the analysis. Coronary artery by-pass grafting surgery was done 24.1% of the ICM patients, all of them more than one year before the SN plasma levels sampling. There was no difference between SN plasma levels in females and males, respectively (27.1 ± 23 vs. 25.5 ± 26.2 pmol/L, *P* = NS); the median and IQR for females/males 26.8 (49.8), 22.4 (41.3) pmol/L, respectively. The ICM vs. DCM group differ in Dyslipidaemia (*P* = 0.003) and creatinine level (*P* = 0.036). The groups did not differ in age, anthropometric factors, diabetes, cerebrovascular accidents, NYHA class, LV EF, analytes, or HF medication (Table 1). Moreover, SN levels were able to discriminate dilated cardiomyopathy from ischemic cardiomyopathy as causation with 88% sensitivity and 61% specificity (AUC 0.73, 95% CI 0.58–0.87, *P* = 0.007, Figure 2), the cut-off value is 13.3 pmol/L. Of note, plasma SN did not vary according to age and was not associated with comorbidities, left ventricular ejection fraction, troponin, creatinine, or natriuretic peptide levels in plasma. There were also no significant correlations between SN and N-terminal pro natriuretic peptide (NT-pro-BNP), high-sensitivity troponin (hs-TnI), creatinine, natrium, total calcium or potassium plasma levels.

There is also no significant difference between total calcium between ICM and DCM (Table 1). However, there is a significant difference in calcium levels between the whole HFrEF group and the healthy individuals (*P* = 0.0001).

Our healthy volunteers (*N* = 34, 62% females, Table 2) have SN plasma levels of 50.7 ± 15.3 pmol/L, which was significantly higher than in both DCM (*P* = 0.049) and ICM (*P* < 0.0001) or HFrEF (ICM/DCM combined), *P* < 0.0001. Males and females in the healthy individuals' group did not differ in any parameters (Table 2). We have not observed myocardial injury in any of the healthy individuals (Table 2). Our healthy volunteers were also significantly younger (*P* < 0.0001) and differed in the BMI (*P* < 0.0001), being lower in the healthy individuals as compared to the HFrEF group.

**Table 2:** Baseline characteristics of the healthy volunteers

	Healthy volunteers	Males	Females	*P* value
	*N* = 34	*N* = 13	*N* = 21	
Age (years)	31 ± 7.1	30.8 ± 7.1	29.9 ± 6.9	0.596
Secretoneurin	50.3 ± 15	50.2 ± 15.1	50.7 ± 15.5	0.64
Body weight (kg)	69.1 ± 13.5	70 ± 14	66.6 ± 13	0.47
Body height (cm)	171.1 ± 8.9	172 ± 8.7	170.6 ± 8.8	0.52
Body mass index (kg/m^2^)	23.2 ± 3.6	23.1 ± 3.6	23 ± 3.6	0.477
NT-pro-BNP	55.4 ± 28.7	58.1 ± 31.4	53.6 ± 28.9	0.066
Creatinine	73 ± 10.9	75.6 ± 10.1	72.3 ± 10.3	0.36
Natrium	138.2 ± 1.73	138 ± 1.8	138.1 ± 1.7	0.208
Potassium	4.1 ± 0.3	4.1 ± 0.29	4.1 ± 0.3	0.03
Ca^2+^ (ionized)	1.17 ± 0.1	1.2 ± 0.1	1.17 ± 0.1	0.519
Total calcium	2.48 ± 0.13	2.48 ± 0.11	2.48 ± 0.13	0.079
Mg^2+^	0.79 ± 0.1	0.79 ± 0.1	0.79 ± 0.05	0.341
hs-TnI	8.6 ± 30	10.2 ± 34.2	8.8 ± 30.1	0.9
Hs-CRP	1.9 ± 2.4	1.5 ± 1.7	2 ± 2.4	0.45
IL-6	3.1 ± 2.6	3.1 ± 2.6	3.2 ± 2.8	0.440
Leukocytes	7.52 ± 1.7	7.5 ± 1.7	7.5 ± 1.6	0.201
Thrombocytes	277.5 ± 59.8	271.3 ± 64	280.4 ± 59.8	0.69

Indices are shown as mean ± standard deviation and compared for males and females. Ca^2+,^ ionized calcium;Hs-TnI, high-sensitivity Troponine I; IL-6, interleukin 6; Mg^2+,^ ionized magnesium; NT-pro-BNP, N-terminal-pro-brain natriuretic peptide.

SN plasma levels in healthy individuals were not associated with total or ionized calcium, hs-CRP, IL-6, pH, hs-TnI, NT-pro-BNP, or albumin level. Plasma levels of SN did not vary according to the age, sex, anthropometric parameters, thrombocytes, haemoglobin, or leukocytes in the healthy individuals (*P* = NS). Potassium and ionized magnesium levels were borderline non-significant when associated to the SN plasma levels (*P* = 0.064, *P* = 0.065, respectively).

## Discussion

### Main findings

The main findings of our preliminary analysis can be summarized as follows:

(1) We found plasma SN levels to be significantly higher in patients with dilated cardiomyopathy than in patients with ischaemic cardiomyopathy.
(2) Plasma SN levels were able to differentiate between DCM and ICM with 88% sensitivity and 61% specificity with a cut-off value 13.3 pmol/L.
(3) Plasma SN levels differed significantly in healthy volunteers as compared to ICM or DCM, healthy volunteers produced higher plasma SN.
(4) Plasma SN levels did not differ according to age or sex and were not associated with comorbidities, left ventricular ejection fraction, troponin, creatinine, or natriuretic peptide levels in plasma.

SN takes part in many processes, namely: apoptosis, immune response, inflammation/chemotaxis, endothelium relaxation, calcium handling, arrhythmogenesis, and cell cycle regulation (2). The broad range of biological effects, independent from classical overload markers such as N-terminal pro-brain natriuretic peptide (NT-pro-BNP) or troponin, suggests that SN is a potentially useful biomarker in cardiology (8). SN has been studied in catecholaminergic polymorphic ventricular arrhythmia, in which it was moderately elevated irrespective of arrhythmogenic episodes (8). SN seems to be a marker reflecting particular cellular mechanism of specific disease than the clinical episodes itself. This pattern of differentiating a type of a disease (ICM vs. DCM), we have observed also in our analysis.

In patients after coronary artery bypass graft (CABG) surgery, SN levels were significantly higher in non-survivors as compared with survivors (173 vs. 143 pmol/L) (9).

The cut-off value for increased mortality risk is >204 pmol/L in patients with aortic stenosis (10). Moreover, in critically ill patients in the intensive care unit, SN was able to predict mortality on top of classical risk factors (11).

In our study, SN levels were somewhat lower than those observed in patients with CABG, critically ill patients, or patients with aortic stenosis before replacement, most of whom were in advanced heart failure or dying (9–11). The reason for lower SN levels in our trial might be the stable state of most of our heart failure patients, who are not comparable to critically ill patients, patients after coronary bypass surgery or younger healthy individuals. On the contrary, in the stable condition of the ICM and/or DCM HFrEF patients are producing even significantly less SN than the healthy individuals in our analysis. Of note, our healthy volunteers were significantly younger but with comparable body size to the HF group.

Moreover, all the previous studies predicted hard clinical endpoints as all-cause or cardiovascular mortality in severe disease states in a completely different patient population compared to our study, differentiating the cause of the disease in stable heart failure.

There are two trials from the same study group evaluating SN in acute and chronic heart failure. In acute heart failure, SN levels were closely associated with mortality. Moreover, SN reclassified patients to their correct risk strata on top of other predictors of mortality (3). In more recent trial in patients with chronic heart failure, SN concentrations were also able to stratify the patients to favourable and poor prognosis on multivariable analysis (5, 12). The SN levels in the chronic HF trial (42;35–62.8 pmol/L) were also very close to our own observations (5, 12).

None of these trials however analysed different SN levels according to the aetiology of HF, ischemic aetiology was only used as an adjusting factor to predict mortality in chronic HF (5, 12). In addition, these trials did not include their own healthy individual subgroup to compare the SN plasma levels with the HF population. Consequently, it is more complicated to draw any conclusion on basal SN secretion patterns in a particular disease specific subgroup of patients. The common denominator of SN elevation in critically ill patients (3, 8–11) is probably tissue ischemia since the studied population otherwise differed in multiple factors. On the contrary, the basal secretion in the fully compensated state may be specific to the underlying disease.

The discriminative capacity of SN with AUC around 0.7 may seem low, though the confidence interval is quite narrow (0.5–0.8). We also must keep in mind that our whole cohort constituted of patients with HFrEF, and no other biomarker has been able to discriminate between the causes of heart failure (ROC curve for hs-TnI and NT-pro BNP are shown in Figure 2). In general, the main goal of biomarkers in heart failure is to provide information regarding the magnitude of risk, and they can also be used to monitor the effects of changes in treatment and detect subclinical disease (13). Although in many of these applications is the utility of SN not clear at the present time, we hope to provide information in the near future on the development of plasma SN levels through time and in response to changes in heart failure treatment. It is becoming clearer, that SN has the capacity to risk stratify and predict the mortality risk in patients with acute and chronic heart failure (3, 5, 12). It is less evident however, whether SN will be able to reflect treatment changes in HF or HF progression. Based on our results SN may also differentiate the ICM vs. DCM cause of heart failure.

We may only speculate that the higher plasma SN levels in DCM, as compared with those in ICM represent more molecularly advanced heart failure and/or impaired calcium handling. Whether higher plasma levels in our study will also predict higher mortality or more frequent heart failure hospitalizations will be revealed in the follow-up. The other, somewhat contradictory, explanation may be that higher plasma SN levels in DCM may be a surrogate for cardiomyocyte's regenerative activity.

Potentially, the SN discriminative capacity (ICM vs. DCM) may be used in the initial heart failure differential diagnosis if confirmed by more extensive trials from different investigator groups.

However, up to now, every elevation of plasma SN levels has led to a worse prognosis in different disease states (8–12). Though all the predictive capacity was limited to acute or critical patient status.

The possible explanation for different SN plasma levels will probably be at the cellular and subcellular levels. In a study with patients with advanced heart failure, ICM and DCM have significantly different molecular profiles (14). DCM (non-ischemic) samples have 32 differentially expressed profiles, and ICM samples have 185 differentially expressed proteins compared to non-failing heart samples (14). The most enriched proteins in ICM are serum amyloid A1, lipopolysaccharide-binding protein, and activated protein C, which are biomarkers associated with coronary artery disease (15, 16). Conversely, non-ischemic enriched proteins are primarily involved in the mitochondrial membrane respiratory chain (14). Also, the extracellular matrix (ECM), a critical component interacting with cells and modulating tissue functions, is different in ICM and DCM (non-ischemic) (14). In DCM, ECM has predominantly interstitial collagen deposition; in ICM, fibrotic replacement is more likely (17). Most interestingly, specific abnormalities in calcium handling have been demonstrated depending on the etiology of HF. ICM is associated with a decreased rate of calcium uptake into the sarcoplasmic reticulum (SR), while DCM is associated with a decreased rate of calcium release from the SR (18).

In our trial, there was no difference in calcium levels between ICM and DCM. However, both groups were hypocalcaemic as compared to healthy individuals. Severe hypocalcaemia may even be the sole cause of heart failure (19). Moreover, in patients after myocardial infarction, low serum calcium may result in a higher mortality rate (20). Extracellular calcium levels may, however not reflect the cytosolic free calcium and related calcium handling in the sarcoplasmic reticulum (21).

All these factors and many unknowns may influence the plasmatic levels of SN in DCM vs. ICM.

Also, our trial confirmed the independence of plasma SN levels from troponin I and NT-pro-BNP levels in plasma, both in HFrEF ICM/DCM patients and healthy individuals. Our results are, at this point, hypothesis-generating at best. Plasma SN levels must be associated with clinical events to show its predictive capacity. Since we have not collected enough clinical events yet, we cannot state whether SN has or has not the prognostic capacity in HFrEF patients. To capture the relationship between calcium handling and SN, the association of ionized calcium plasma levels may be sampled and correlated with SN plasma levels in future trials.

### Limitations

Our sample was small and unbalanced for sex, with male predominance in the heart failure group and female predominance in the healthy volunteer group. Moreover, there were more patients with ischemic cardiomyopathy than with dilated cardiomyopathy. These results cannot be extrapolated to different patient population (different cardiomyopathies) or acute state of the heart failure.

## Conclusion

Plasma secretoneurin levels differed significantly in DCM vs. ICM, being higher in the former. Moreover, discrimination between DCM and ICM might be possible based on plasma SN levels. Healthy individuals produce higher SN plasma levels than stable HFrEF patients.

## Data Availability

The raw data supporting the conclusions of this article will be made available by the authors upon reasonable request and in compliance with the General Data Protection Rule (GDPR).

## References

[1] Anderson ME. Will secretoneurin be the next big thing. J Am Coll Cardiol. (2015) 65(4):352–4. 10.1016/j.jacc.2014.11.028 25634833

[2] Plášek J Lazárová M Dodulík J Šulc P Stejskal D Švagera Z Secretoneurin as a novel biomarker of cardiovascular episodes: are we there yet? A narrative review. J Clin Med. (2022) 11(23):7191. 10.3390/jcm11237191 36498765 PMC9735894

[3] Ottesen AH Louch WE Carlson CR Landsverk OJB Kurola J Johansen RF Secretoneurin is a novel prognostic cardiovascular biomarker associated with cardiomyocyte calcium handling. J Am Coll Cardiol. (2015) 65:339–51. 10.1016/j.jacc.2014.10.065 25634832

[4] Luo M Anderson ME. Mechanisms of altered Ca^2+^ handling in heart failure. Circ Res. (2013) 113(6):690–708. 10.1161/CIRCRESAHA.113.301651 23989713 PMC4080816

[5] Røsjø H Meessen J Ottesen AH Latini R Omland T, GISSI HF investigators. Prognostic value of secretoneurin in chronic heart failure. Data from the GISSI-heart failure trial. Clin Biochem. (2023) 118:110595. 10.1016/j.clinbiochem.2023.110595 37277028

[6] Aakre KM Ottesen AH Strand H Faaren AL Alaour B Torsvik J Biologial variation of secretoneurin; a novel cardiovascular biomarker implicated in arrhythmogenesis. Clin Biochem. (2021) 98:74–7. 10.1016/j.clinbiochem.2021.09.014 34624255

[7] Dodulík J Václavík J Lazarová M Šulc P Evin L Stejskal D Secretoneurin plasma levels in patients with different etiologies of heart failure: preliminary data—abstracts of the heart failure 2023 and the world congress on acute heart failure, 20–23 May 2023, Prague, Czechia. Eur J Heart Fail. (2023) 25(S2):144. 10.1002/ejhf.2927

[8] Ottesen AH Carlson CR Eken OS Sadredini M Myhre PL Shen X Secretoneurin is an endogenous CaMKII inhibitor that attenuates Ca^2+^-dependent arrhythmia. Circ Arrhythm Electrophysiol. (2019) 12:007045. 10.1161/CIRCEP.118.007045 30943765

[9] Brynildsen J Petäjä L Myhre PL Lyngbakken MN Nygård S Stridsberg M Circulating secretoneurin concentrations after cardiac surgery: data from the FINNish acute kidney injury heart study. Crit Care med. (2019) 47(5):e412–9. 10.1097/CCM.0000000000003670 30730440

[10] Brynildsen J Myhre PL Lynbakken MN Klaeboe LG Sridsberg M Christensen G Circulating secretoneurin concentrations in patients with moderate to severe aortic stenosis. Clin Biochem. (2019) 71:17–23. 10.1016/j.clinbiochem.2019.06.008 31228433

[11] Røsjø H Stridsberg M Ottesen AH Nygård S Christensen G Pettilä V Prognostic value of secretoneurin in critically ill patients with infections. Crit Care. (2016) 44(10):1882–90. 10.1097/CCM.0000000000001832 27414477

[12] Rosjo H Meessen J Ottesen AH Omland H, GISSI-HF study. Circulating secretoneurin concentrations provide independent prognostic information to established risk indices in patients with chronic heart failure. Eur Heart J. (2022) 43(2):ehac544.912. 10.1093/eurheartj/ehac544.912

[13] Miller WL Jaffe AS. Biomarkers in heart failure: the importance of inconvenient details. ESC Heart Fail. (2016) 3(1):3–10. 10.1002/ehf2.12071 27774262 PMC5063139

[14] Zhao Y Godier-Furnemont A Bax NAM Bouten CVC Brown LM Vunjak-Novakonic G. Changes in extracellular matrix in failing human non-ischemic nad ischemic hearts with mechanical unloading. J Mol Cell Cardiol. (2022) 166:137–51. 10.1016/j.yjmcc.2022.02.003 35219725 PMC9035113

[15] Kosuge M Ebina T Ishikawa T Hibi K Tsukara K Okuda J Serum amyloid A is a better predictor of clinical outcomes than C-reative protein in non-ST-segment elevation acute coronary syndromes. Circ J. (2007) 71(2):186–90. 10.1253/circj.71.186 17251664

[16] Lepper PM Kleber ME Grammer TB Hoffmann K Dietz S Winkelmann BR Lipopolysaccharide-binding protein (LBP) is associated with total and cardiovascular mortality in individuals with or without stable coronary artery disease—results from the ludwigshafen risk and cardiovascular health study (LURIC). Atherosclerosis. (2011) 219(1):291–7. 10.1016/j.atherosclerosis.2011.06.001 21722903

[17] Frangogiannis NG. The extracellular matrix in ischemic and nonischemic heart failure. Circ Res. (2019) 125(1):117–46. 10.1161/CIRCRESAHA.119.311148 31219741 PMC6588179

[18] Sen L Cui G Fonarow GS Laks H. Differences in mechanisms of SR dysfunction in ischemic vs. idiopathic dilated cardiomyopathy. Am J Physiol Heart Circ Physiol. (2000) 279(2):H709–18. 10.1152/ajpheart.2000.279.2.H709 10924070

[19] Baqi DH Ahmed SF Baba HO Fattah FH Salih AM Ali RM Hypocalcemia as a cause of reversible heart failure: a case report and review of the literature. Ann Med Surg. (2022) 77:103572. 10.1016/j.amsu.2022.103572 PMC9142408 35637983

[20] Schmitz T Thilo Ch Linseisen J Heier M Peters A Kuch B Low serum calcium is associated with higher long-term mortality in myocardial infarction patients from a population-based registry. Sci Rep. (2021) 11(1):2476. 10.1038/s41598-021-81929-7 33510279 PMC7843683

[21] Barbagallo M Dominguez LJ Licata G Resnick LM. Effects of aging on serum ionized and cytosolic free calcium: relation to hypertension and diabetes. Hypertension. (1999) 34(2):902–6. 10.1161/01.HYP.34.4.902 10523382

