# Corrigendum: Secretoneurin levels are higher in dilated cardiomyopathy than in ischaemic cardiomyopathy: preliminary results

**DOI:** 10.3389/fcvm.2024.1392484

**Published:** 2024-04-03

**Authors:** Jiří Plášek, Jozef Dodulík, Marie Lazárová, David Stejskal, Zdeněk Švagera, Nela Chobolová, Patrik Šulc, Lukáš Evin, Dana Purová, Jan Václavík

**Affiliations:** ^1^ Department of Internal Medicine and Cardiology, University Hospital Ostrava, Ostrava, Czechia; ^2^ Research Center for Internal and Cardiovascular Diseases Faculty of Medicine, University of Ostrava, Ostrava, Czechia; ^3^ Institute of Laboratory Medicine, University Hospital Ostrava, Ostrava, Czechia; ^4^ Institute of Laboratory Medicine, University of Ostrava, Ostrava, Czechia; ^5^ Social Health Institute, Palacky University Olomouc, Olomouc, Czechia

**Keywords:** secretoneurin, heart failure, CaMKII, dilated cardiomyopathy, ischaemic cardiomyopathy

A Corrigendum on Secretoneurin levels are higher in dilated cardiomyopathy than in ischaemic cardiomyopathy: preliminary results By Plášek J, Dodulík J, Lazárová M, Stejskal D, Švagera Z, Chobolová N, Šulc P, Evin L, Purová D and Václavík J. (2023). Front. Cardiovasc. Med. doi: 10.3389/fcvm.2023.1297900

In the published article, there were multiple errors in Table 2 as published. The values in Table 2 as seen below have been corrected.

**Table 2:** Baseline characteristics of the healthy volunteers.

	Healthy volunteers	Males	Females	*P* value
	*N* = 34	*N* = 13	*N *= 21	
Age (years)	31 ± 7.1	30.8 ± 7.1	29.9 ± 6.9	0.596
Secretoneurin	50.7 ± 16.3	50.7 ± 15.3	50.7 ± 16.3	0.64
Body weight (kg)	69 ± 14	69 ± 14	67 ± 14	0.001
Body height (cm)	171.8 ± 8.9	171.8 ± 8.9	170.8 ± 8.9	<0.001
Body mass index (kg/m^2^)	23.2 ± 3.6	23.1 ± 3.6	23 ± 3.6	0.477
NT-pro-BNP	55.5 ± 29.2	55.5 ± 29.2	57.5 ± 30	0.066
Creatinine	74.1 ± 12.45	74.1.1 ± 12.45	71.9 ± 10.6	<0.001
Natrium	138.2 ± 1.75	138.2 ± 1.75	138.2 ± 1.76	0.208
Potassium	4.1 ± 0.29	4.1 ± 0.29	4.1 ± 0.3	0.03
Ca^2+^ (ionized)	1.17 ± 0.1	1.2 ± 0.1	1.17 ± 0.1	0.519
Total calcium	2.48 ± 0.13	2.48 ± 0.11	2.48 ± 0.13	0.079
Mg^2+^	0.79 ± 0.1	0.79 ± 0.1	0.79 ± 0.05	0.341
hs-TnI	8.1 ± 29.4	8.1 ± 29.42	8.6 ± 31	0.195
Hs-CRP	1.8 ± 2.36	1.8 ± 2.4	2 ± 2.4	0.026
IL-6	3.1 ± 2.6	3.1 ± 2.6	3.2 ± 2.8	0.440
Leukocytes	7.52 ± 1.7	7.5 ± 1.7	7.5 ± 1.6	0.201
Thrombocytes	280 ± 59.1	280 ± 59.1	281.8 ± 59	0.013

Indices are shown as mean ± standard deviation and compared for males and females. Ca^2+^, ionized calcium; Hs-TnI, high-sensitivity Troponine I; IL-6, interleukin 6; Mg^2+^, ionized magnesium; NT-pro-BNP, N-terminal-pro-brain natriuretic peptide.

The corrected Table 2 and its caption appear below.

**Table 2:** Baseline characteristics of the healthy volunteers.

	Healthy volunteers	Males	Females	*P* value
	*N* = 34	*N* = 13	*N* = 21	
Age (years)	31 ± 7.1	30.8 ± 7.1	29.9 ± 6.9	0.596
Secretoneurin	50.3 ± 15	50.2 ± 15.1	50.7 ± 15.5	0.64
Body weight (kg)	69.1 ± 13.5	70 ± 14	66.6 ± 13	0.47
Body height (cm)	171.1 ± 8.9	172 ± 8.7	170.6 ± 8.8	0.52
Body mass index (kg/m^2^)	23.2 ± 3.6	23.1 ± 3.6	23 ± 3.6	0.477
NT-pro-BNP	55.4 ± 28.7	58.1 ± 31.4	53.6 ± 28.9	0.066
Creatinine	73 ± 10.9	75.6 ± 10.1	72.3 ± 10.3	0.36
Natrium	138.2 ± 1.73	138 ± 1.8	138.1 ± 1.7	0.208
Potassium	4.1 ± 0.3	4.1 ± 0.29	4.1 ± 0.3	0.03
Ca^2+^ (ionized)	1.17 ± 0.1	1.2 ± 0.1	1.17 ± 0.1	0.519
Total calcium	2.48 ± 0.13	2.48 ± 0.11	2.48 ± 0.13	0.079
Mg^2+^	0.79 ± 0.1	0.79 ± 0.1	0.79 ± 0.05	0.341
hs-TnI	8.6 ± 30	10.2 ± 34.2	8.8 ± 30.1	0.9
Hs-CRP	1.9 ± 2.4	1.5 ± 1.7	2 ± 2.4	0.45
IL-6	3.1 ± 2.6	3.1 ± 2.6	3.2 ± 2.8	0.440
Leukocytes	7.52 ± 1.7	7.5 ± 1.7	7.5 ± 1.6	0.201
Thrombocytes	277.5 ± 59.8	271.3 ± 64	280.4 ± 59.8	0.69

Indices are shown as mean ± standard deviation and compared for males and females. Ca^2+^, ionized calcium; Hs-TnI, high-sensitivity Troponine I; IL-6, interleukin 6; Mg^2+^, ionized magnesium; NT-pro-BNP, N-terminal-pro-brain natriuretic peptide.

The authors apologize for this errors and state that this does not change the scientific conclusions of the article in any way. The original article has been updated.

In the published article, there was an error in assumptions about differences in males and females in the healthy volunteers group.

A correction has been made to **Results**. This sentence previously stated:

“Males and females in the healthy individuals group differed in anthropometric parameters, plasma creatinine level, hs-CRP, and thrombocyte count (Table 2)”.

The corrected sentence appears below:

“Males and females in the healthy individuals’ group did not differ in any parameters (Table 2)”.

The authors apologize for this error and state that this does not change the scientific conclusions of the article in any way. The original article has been updated.

